# A neuroligin-1-derived peptide stimulates phosphorylation of the NMDA receptor NR1 subunit and rescues MK-801-induced decrease in long-term potentiation and memory impairment

**DOI:** 10.1002/prp2.126

**Published:** 2015-03-13

**Authors:** Irina Korshunova, Michelle D Gjørlund, Sylwia Owczarek, Anders V Petersen, Jean-François Perrier, Casper René Gøtzsche, Vladimir Berezin

**Affiliations:** 1 Laboratory of Neural Plasticity, University of CopenhagenCopenhagen, Denmark; 2 Neuronal Signaling Laboratory, Department of Neuroscience and Pharmacology, University of CopenhagenCopenhagen, Denmark

**Keywords:** Learning and memory, LTP, MK-801, neuroligin, NMDA receptor

## Abstract

Neuroligins (NLs) are postsynaptic adhesion molecules, interacting with presynaptic neurexins (NXs), which determine the differential formation of excitatory (glutamatergic, NL1) and inhibitory (GABAergic, NL2) synapses. We have previously demonstrated that treatment with a NL2-derived peptide, neurolide-2, reduces sociability and increase animal aggression. We hypothesized that interfering with NL1 function at the excitatory synapses might regulate synaptic plasticity and learning, and counteract memory deficits induced by *N*-methyl-d-aspartate (NMDA) receptor inhibition. First, neuronal NMDA receptor phosphorylation after treatment with NL1 or a mimetic peptide, neurolide-1, was quantified by immunoblotting. Subsequently, we investigated effects of neurolide-1 on long-term potentiation (LTP) induction in hippocampal slices compromised by NMDA receptor inhibitor MK-801. Finally, we investigated neurolide-1 effects on short- and long-term social and spatial memory in social recognition, Morris water-maze, and Y-maze tests. We found that subcutaneous neurolide-1 administration, restored hippocampal LTP compromised by NMDA receptor inhibitor MK-801. It counteracted MK-801-induced memory deficit in the water-maze and Y-maze tests after long-term treatment (24 h and 1–2 h before the test), but not after short-term exposure (1–2 h). Long-term exposure to neurolide-1 also facilitated social recognition memory. In addition, neurolide-1-induced phosphorylation of the NMDA receptor NR1 subunit on a site important for synaptic trafficking, potentially favoring synaptic receptor retention. Our findings emphasize the role of NL1–NMDA receptor interaction in cognition, and identify neurolide-1, as a valuable pharmacological tool to examine the in vivo role of postsynaptic NL1 in cognitive behavior in physiological and pathological conditions.

## Introduction

Neuroligins (NLs) are synaptic adhesion molecules that determine the differential formation of excitatory (glutamatergic, NL1) and inhibitory (*γ*-aminobutyric acid-ergic, NL2) synapses. Various mutations in NL genes have been identified in individuals with autism (Sudhof 2008). The rodent genome encodes four NL isoforms, NL1-4. NLs are expressed at postsynaptic sites as dimers that interact with presynaptically expressed neurexins (NXs). Upon synapse formation, the ligation of NL1 by NXs induces the recruitment of postsynaptic density protein 95, synaptic scaffolding molecule, and, indirectly, *N*-methyl-d-aspartate (NMDA) and *α*-amino-3-hydroxy-5-methyl-4-isoxazolepropionic acid (AMPA) glutamate receptors (Krueger et al. 2012). The ectodomain of NLs contains two sequence segments that can be alternatively spliced: insert A (NL1-3) and insert B (uniquely NL1; [Arac et al. 2007; Chen et al. 2008]). The B insert determines strong interactions with a short NX1 isoform, namely NX-1*β*, that lacks splice site SS4. Splice site SS4 is localized at the edge of the NL-binding interface and, to some extent, the opposite insert B of NL1 (Arac et al. 2007). The SS4 region forms a long *α*-helix that is thought to interfere with a salt bridge that is known to be important for NX–NL interactions. NXs that contain SS4 have been shown to promiscuously bind various NLs, whereas NX-1*β* that lacks SS4 interacts predominantly with NL1 that expresses the B insert (Shen et al. 2008). The expression of the B insert is specific for excitatory synapses, and it may also play a role in modulating the function in NX–NL complexes at synapses (Krueger et al. 2012).

In the hippocampus, NL1 regulates excitatory synaptic transmission (Jedlicka et al. 2013), and its overexpression leads to an increase in synaptic excitation, which impairs synaptic plasticity and promotes learning deficits (Dahlhaus et al. 2010). The NL1 ectodomain has been shown to make *cis*-interactions with the NMDA GluN NR1 receptor subunit, which is regarded as a key mechanism in controlling the properties of glutamatergic synapses by promoting synaptic incorporation and the retention of NMDA receptors (Budreck et al. 2013). NR1 phosphorylation on serine 897, a protein kinase A (PKA) phosphorylation site, has been suggested to increase the trafficking of NMDA receptors to the cell surface in neurons (Scott et al. 2001).

We recently investigated the functional role of the NL1 B insert using a peptide, termed neurolide-1, that encompasses the NL1-binding site for NX1, including the B insert. Neurolide-1 was shown to bind NX-1*β* and, in contrast to the peptide without the B insert, induce neurite outgrowth in hippocampal neurons in an NX-1*β* expression-dependent manner (Gjorlund et al. 2012). Another study investigated in vivo intra-hippocampal treatment with a peptide, termed neurolide-2, derived from NL2 without the B insert and found that it decreased sociability and increased aggression, thus mimicking the effect of chronic restraint stress in adulthood, whereas neurolide-1 did not demonstrate such effects (van der Kooij et al. 2014). Chronic restraint stress has been shown to reduce NL2 but not NL1 levels in different layers of the hippocampus. Therefore, interfering with NL2 but not NL1 function appears to alter social and aggressive behavior (van der Kooij et al. 2014).

In the present study, we investigated the pharmacological properties of neurolide-1 in vitro and in vivo. We hypothesized that the NL1 ectodomain or neurolide-1 could be used to interfere with cognate NL1 function at excitatory synapses, specifically with NL1 that contains the B insert, and this might improve synaptic plasticity and learning performance, especially when these are compromised by NMDA receptor inhibition. Using a combination of biochemical, electrophysiological, and behavioral approaches, we obtained results that supported our hypothesis.

## Materials and Methods

### Legal and ethical guidelines for use of animals

All experiments involving animals, including preparation of primary neuronal cultures, acute slice preparation and in vivo studies, followed the ARRIVE guidelines, were performed in accordance with Danish Animal Welfare Agency guidelines under directive BEK 88 (30 January 2013), European Union directive 2010/63/EU (22 September 2010) on the protection of animals used for scientific purposes and were approved by the local Ethical Committee for Experimental Animals. All animals were group-housed under standard conditions (23°C, 50% humidity, 12/12 h light/dark cycle) with wood shavings, bedding, a shelter, free access to food and water, and general health and wellbeing of the animals were assessed regularly by veterinarians and trained caretakers. Every effort was made to minimize the number of animals used and to minimize any distress. Rats were euthanized by concussive stunning rendering them unconscious follow by cervical dislocation, a method regarded as acceptable and humane, since it has the advantage of being highly reliable, rapid, independent of drug pharmacokinetics and presumably induces minimal pain, distress, fear, and anxiety since it requires little preparatory handling. Mice were rapidly and humanely euthanized with a minimum of distress by decapitation without preceding anesthetic. Importantly, it leaves the tissue free of pharmacologically active drug residues. Appropriately trained and experienced personnel performed all animal handling and euthanization.

### Peptides and recombinant proteins

The neurolide-1 peptide (amino acid sequence: SEGNRWSNSTKGLFQRA) was synthesized by Schafer-N (Copenhagen, Denmark) as a tetramer composed of four monomers coupled to a lysine backbone (Gjorlund et al. 2012). The rat recombinant NL1 ectodomain was obtained from R&D Systems (Abingdon, UK).

### NMDA receptor phosphorylation assay

Cortical neurons were obtained from Wistar rat embryos at embryonic day 19, of both sexes, basically as previously described (Maar et al. 1997) (see Data S1). Briefly, animals were rendered unconscious by concussive stunning and euthanized by cervical dislocation. Hereafter, the embryonic brains were collected and cortical neurons were dissociated and seeded in 60-mm tissue culture dishes coated with 1 *μ*g/mL of poly-d-lysine (Sigma-Aldrich, St. Louis, MO) and grown for 8 days before treatment. Then, the cultures were stimulated with either the NL1 ectodomain (0.2, 0.6, 1.8, and 5.4 nmol/L) or neurolide-1 (0.1, 0.3, 1, and 2.7 *μ*mol/L) for 5 min. The cells were lysed, and proteins were resolved by sodium dodecyl sulfate-polyacrylamide gel electrophoresis followed by immunoblotting using rabbit anti-Ser897-NR1 antibody (1:500; Millipore, Billerica, MA), mouse anti-*β*-tubulin antibody (1:50,000; Sigma-Aldrich), and IRDye secondary antibodies (Odyssey, Lincoln, NE). The bands were visualized using the Odyssey CLX Infrared Imaging System (Odyssey). Four independent experiments with stimulation by NL1 ectodomain and eight independent experiments with stimulation by neurolide-1 were performed.

### Mouse hippocampus Long-term potentiation protocol

#### Slice preparation

Juvenile *C57BL/6N* mice of both sexes (postnatal day 16–24; Taconic, Ejby, Denmark) were injected subcutaneously (s.c.) with 10 mg/kg of the neurolide-1 peptide solution (10 mg/mL) and again 23 h later with 10 mg/kg of the neurolide-1 peptide solution (10 mg/mL) and 0.1 mg/kg MK-801 solution (0.025 mg/mL). Mice receiving the injection with the MK-801 solution only or untreated mice served as control groups. The young mice were rapidly and humanely euthanized with a minimum of distress by decapitation at a time point corresponding to 24 h after first injection. Preceding anesthetic was omitted to preserve the tissue free of drug residues and brain slice functions as unaltered as possible when performing the physiological measurements. After decapitation, the brain was removed. Parasagittal slices (300 *μ*m) from the left hemisphere were cut on a vibratome (MicroM HM 650V, Microm, Bicester, UK) equipped with a CU65 cooling unit while the tissue was immersed in cooled (2°C) artificial cerebrospinal fluid (aCSF) of the following composition: 125 mmol/L NaCl, 2.5 mmol/L KCl, 26 mmol/L NaHCO_3_, 1.25 mmol/L NaH_2_PO_4_·H_2_O, 1 mmol/L MgCl_2_, 2 mmol/L CaCl_2_, and 25 mmol/L glucose, bubbled with 5% CO_2_ in 95% O_2_. The slices rested in oxygenated aCSF (35°C) for at least 1 h before measurements were made. In the study, *n* refers to number of animals and each animal contributed with only one slice.

#### Long-term potentiation

Measurements were made in oxygenated aCSF at room temperature (1.1 mL/min). Schaeffer collaterals were stimulated with a bipolar concentric electrode (TM33CCNON; World Precision Instruments, Sarasota, FL). The field potential in the stratum radiatum of the CA1 area of the hippocampus was recorded with an extracellular glass microelectrode (4–6 MΩ, filled with aCSF), positioned at least 500 *μ*m away from the stimulation electrode. The stimulus intensity was set 0.03 mA above the threshold. After a 30-min baseline was obtained with stimulation at 0.05 Hz, long-term potentiation (LTP) was induced by stimulating the Schaeffer collaterals at 100 Hz for 1 s four times at 20 s intervals. A new baseline was established over the following 30 min. Potentiation was estimated by comparing the rising slope of the field excitatory postsynaptic potential (fEPSP) before and 25–30 min after high-frequency stimulation.

### Drug treatment

For animal experiments the neurolide-1 peptide or vehicle (phosphate-buffered saline; PBS) was injected s.c. (10 mg/kg) in a volume of 1 mL/kg body weight. The NMDA receptor antagonist [1]-5-methyl-10,11-dihydro-5H-dibenzo-[a,d]-cyclohepten-5,10-imine hydrogen maleate (MK-801; Sigma-Aldrich, Brøndby, Denmark) was dissolved in PBS. MK-801 (0.1 mg/kg) or vehicle was injected s.c. in a volume of 1 mL/kg body weight. This MK-801 dose has been reported to impair cognition in rats without causing significant sensory, locomotor, or toxicological side effects and reaches a sufficient level of extracellular fluid concentrations in the brain to disrupt NMDA receptor function (Brosnan-Watters et al. 1996; Andine et al. 1999).

### Pharmacokinetics

Biotinylated neurolide-1 (Schafer-N) was administered s.c. to male Wistar rats weighing 200–250 g (see Data S1). Blood samples (300 *μ*L) were collected from rats at time points 0 min, 15 min, 1 h, 4 h, and 24 h or 0 min, 30 min, 2 h, and 8 h after peptide administration from the orbital plexus under isoflurane anesthesia (*n *=* *2 in each group). Cerebrospinal fluid (CSF) was collected in rats (*n *=* *6 in each group) under fentanyl/droperidol/midazolam anesthesia (0.002%/0.14%/0.014% w/v) from the cisterna magna 30 min after peptide administration as described previously (Secher et al. 2006). Rats for blood and CSF samples were euthanized under anesthesia by cervical dislocation after last sampling. Plasma and CSF samples were stored at −80°C. The levels of neurolide-1 in plasma and CSF samples were measured using a competitive enzyme-linked immunosorbent assay as described previously (Klementiev et al. 2014).

### Social recognition test

Social memory in animals reflects the ability to recognize conspecifics as familiar or unfamiliar. The social recognition test was performed basically as described previously (Secher et al. 2006), (see Data S1). Briefly, adult male Wistar Rats (150–200 g on the day of arrival; Charles River, Sulzfeld, Germany) were given two s.c. injections of Neurolide-1 or vehicle at time points 24 and 2 h before the test (*n *=* *12 in all groups). Rats were handled 5 days prior to the experiment and habituated to the transparent test cages for 1 h before start of experiment. The tests included three trials (T1–3), separated by intertribal periods of 2 and 24 h, with the test rat being introduced to a 3-week-old juvenile conspecific (40–50 g; Charles River) for 4 min each time. Investigative behavior was recorded and used to calculate recognition ratios (RR) as previously described (Kogan et al. 2000). Retention periods longer than 1 h are believed to result in no or only minimal retention of social memory (Popik and van Ree 1998; Secher et al. 2006). Terminally, rats were rapidly and humanely rendered unconscious by concussive stunning and euthanized by cervical dislocation.

### Spatial memory in the Morris water maze

The Morris water-maze test (Morris 1984) was performed as previously described (Kraev et al. 2011) with slight modifications (see Data S1). Male Wistar rats (Charles River), weighing 150–200 g on the day of arrival were used. To investigate for potential effects on memory consolidation, neurolide-1 was injected immediately after the training sessions on day 1 and 2 (*n *=* *12 in each group). To test for potential effects on memory acquisition neurolide-1 was injected 2 h before start of training on training day 1 and 2 (*n *=* *12 in each group). Finally, to test for potential effects on MK-801-induced learning deficiencies neurolide-1 was injected 2 h before start of training on day 2 and 3 in animals, which had received an injection with MK-801 30 min earlier (*n *=* *6–9 in each group). In a slightly modified version of the MK-801 experiments a group of animals received a neurolide-1 injection immediately after the training sessions on day 1, and then a neurolide-1 injection 2 h before start of training on day 2, preceded by an MK-801 injection 30 min earlier (*n *=* *12 in each group). Terminally, rats were rapidly and humanely rendered unconscious by concussive stunning and euthanized by cervical dislocation.

### Y maze test

NMDA-mediated effects of Neurolide-1 on spatial working memory performance was assessed by recording spontaneous alternation behavior in a Y maze test performed as described previously (Wegener et al. 2011), (See Data S1). Initially, adult male Wister rats (420–490 g, *n *=* *6–12; Charles River) were injected s.c., three times at 28, 4, and 2 h before start of the Y maze test. One group received Neurolide-1, saline, and neurolide-1 in this order. Another group received saline, MK-801, and saline in this order. A third group received neurolide-1, MK-801, and neurolide-1 in this order. And control group received saline at all times. In a second setup we tested the effect of neurolide-1 in very young male Wistar rats (4-weeks old) which received a s.c., injection of neurolide-1 or saline 2 h prior to testing them in the Y-maze (*n *=* *11–15). Terminally, rats were rapidly and humanely rendered unconscious by concussive stunning and euthanized by cervical dislocation.

### Data analysis

The statistical analysis was performed using one-way analysis of variance (ANOVA) followed by the Newman–Keuls multiple-comparison post hoc or Dunnett tests, Mann–Whitney *U*-test, or one-sample *t*-test when appropriate (GraphPad Software, La Jolla, CA, USA, www.graphpad.com).

## Results

### Neurolide-1 induces phosphorylation of the NR1 subunit on serine 897 in cortical neurons

**Figure 1:**
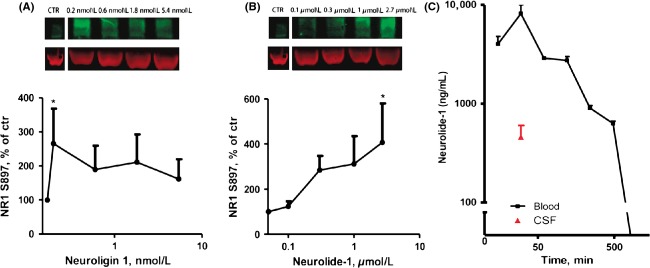
Effects of recombinant NL1 and neurolide-1 on phosphorylation of the NMDA receptor NR1 subunit. The figure shows Western blots of extracts from cultured cortical neurons treated with various concentrations of NL1 (A) and neurolide-1 (B) with anti-phospho-Ser897-NR1 antibody. The data are expressed as mean ± SEM. *P <* *0.05 (one-way ANOVA followed by Dunnett's post hoc test). (C) Time-course of neurolide-1 concentration in plasma (0 min, 15 min, 30 min, 1 h, 2 h, 4 h, 8 h and 24 h) and CSF (30 min) after a single subcutaneous injection of biotin-labeled peptide (10 mg/kg). The data are expressed as mean ± SEM (*n *=* *2 for blood samples, *n *=* *6 for CSF samples). NMDA, *N*-methyl-D-aspartate; ANOVA, analysis of variance; CSF, cerebrospinal fluid.

The ectodomain of NL1 has been shown to directly interact with the NMDA receptor NR1 subunit (Budreck et al. 2013), which has a significant impact on postsynaptic signaling, including the facilitation of synaptic receptor retention and trafficking. The phosphorylation of NR1 on serine 897 by PKA is suggested to increase NR1 trafficking to the cell surface (Scott et al. 2001). We studied whether the NL1 ectodomain induces NR1 phosphorylation using specific antibodies that recognize NL1 phosphorylation at serine 897. NL1-induced NR1 phosphorylation when added to cultured cortical neurons at a concentration as low as 0.1 nmol/L. At higher concentrations, NL1 did not have a statistically significant effect on NR1 phosphorylation (Fig.1A). The peptide that encompasses the minimal binding site of NL1 for NX-1*β*, neurolide-1, induced NR1 phosphorylation proportionally to the increase in peptide concentration (Fig.1B).

### Neurolide-1 crosses the blood-brain barrier

To study the effect of systemic administration of neurolide-1 on cognitive function in animals, we investigated whether it crosses the blood-brain barrier. The study was performed using biotinylated neurolide-1. Neurolide-1 (10 mg/kg) was detectable in plasma 15 min after s.c. administration (4025 ± 785 ng/mL) and remained detectable for up to 8 h (633 ± 33 ng/mL). Detection peaked 30 min after administration, and it was also present in CSF at this time point (460 ± 143 ng/mL; Fig.1C). The plasma/CSF concentration ratio was ∼20. Thus, neurolide-1 crossed the blood-brain barrier after systemic administration.

### Neurolide-1 restores LTP in acute hippocampal slices compromised by the NMDA receptor inhibitor MK-801

**Figure 2:**
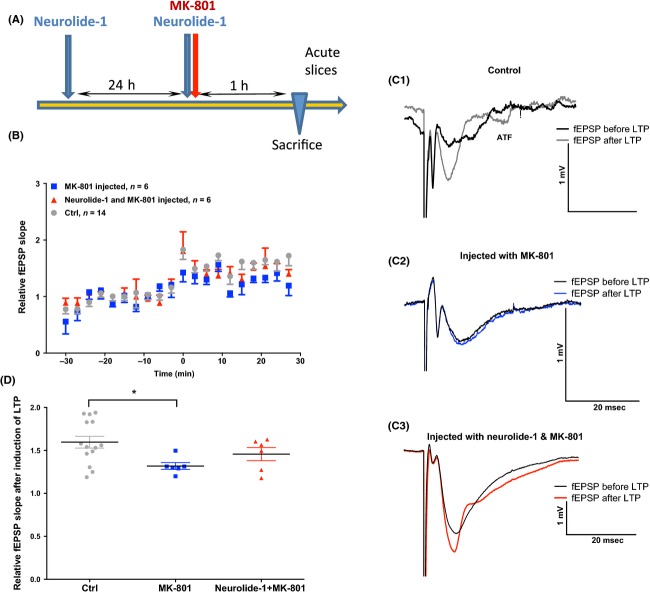
Effect of subcutaneously injected neurolide-1 (24 h and 1 h before sacrifice) on LTP in acute hippocampal slices. (A) Timeline of the experimental paradigm. (B) Plot of normalized fEPSP slope evoked at the CA3–CA1 synapse over time in hippocampal slices from untreated mice (grey circles), mice injected with either MK-801 alone (blue squares), or mice treated with MK-801 combined with neurolide-1 (red triangles). High-frequency stimulation (HFS) was applied at time zero. (C1–C3) fEPSP slopes from the different conditions 5 min before and 25 min after LTP induction. (D) Relative changes in fEPSP slope induced by LTP. The data are expressed as mean ± SEM. *P <* *0.05 (one-way ANOVA followed by Newman–Keuls post hoc test). LTP, long-term potentiation; fEPSP, field excitatory postsynaptic potential; ANOVA, analysis of variance.

LTP is the long-lasting enhancement of synaptic efficacy induced by the high-frequency stimulation of afferent fibers and considered one of the major cellular mechanisms that underlie learning and memory (Shors and Matzel 1997). One form of LTP is caused by an increase in the number of functional postsynaptic NMDA receptors. We investigated whether neurolide-1 rescues hippocampal LTP impairment induced by the noncompetitive NMDA receptor antagonist MK-801. In untreated mice, the fEPSP slope increased by 64 ± 7% (Fig.2A and B, c1, *n* = 14). Pretreatment with MK-801 significantly inhibited the induction of LTP (Fig.2D, c2; 27 ± 5% increase; *n *=* *6; *P *=* *0.03, one-way ANOVA followed by Newman–Keuls multiple-comparisons test). In contrast, in mice injected with both neurolide-1 and MK-801, a 57 ± 12% increase was observed. The relative fEPSP slope in this group was not different from the control group, indicating that neurolide-1 can partially restore LTP that is compromised by MK-801 (Fig.2D, c3; *n *=* *6; one-way ANOVA followed by Newman–Keuls multiple-comparison test). The direct treatment of hippocampal slices with neurolide-1 alone did not affect LTP (Fig. S1).

### Neurolide-1 facilitates social recognition memory

Social memory refers to the ability of animals to change their social behavior toward a conspecific as a consequence of the memory of a previous social encounter. Social recognition memory in rodents appears to be based mainly on olfactory cues (Popik and van Ree 1998; Young 2002). Social recognition in rats is based on the natural tendency to intensively investigate unfamiliar individuals. Memory is measured as the decrease in the time that adult rats spend investigating a juvenile during a second encounter. The retention of social memory decreased significantly when the interval between the tests increased. After ∼1–2 h, an adult rat loses short-term memory of a presented juvenile (Secher et al. 2006).

**Figure 3:**
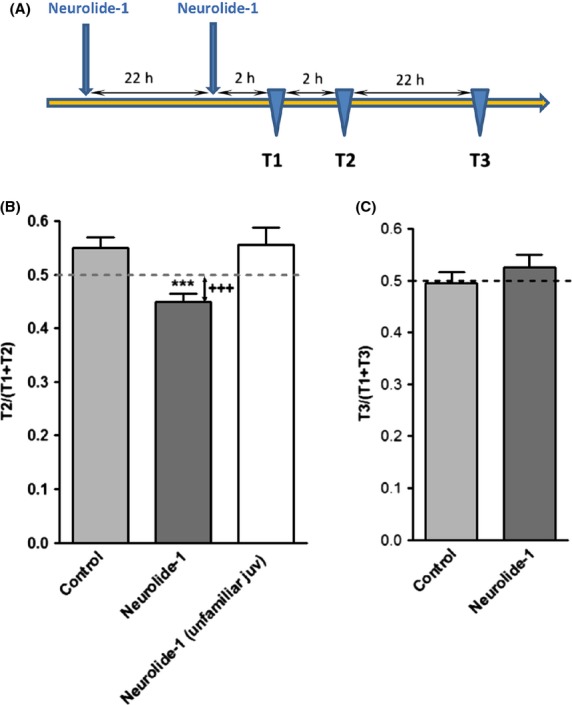
Effect of neurolide-1 on social recognition memory. (A) Timeline of the experimental paradigm. The figure shows the social memory recognition ratio with intertrial intervals of 2 h (B) or 24 h (C). The data are expressed as mean ± SEM (*n *=* *12 per group). ***P <* *0.001, compared with control (one-way ANOVA followed by Newman–Keuls post hoc test); ^+++^*P *<* *0.001, compared with the hypothetical value 0.5 (one-sample *t*-test). ANOVA, analysis of variance.

To test whether neurolide-1 augments social memory formation and facilitates retention, we used an intertrial interval of 2 h to evaluate short-term social memory and 24 h to evaluate long-term social memory (Fig.3A).

The animals received 10 mg/kg neurolide-1 s.c. both 24 and 1 h before the first trial. Neurolide-1-enhanced social memory (Fig.3B) when the intertrial interval between the two trials was 2 h (*P < *0.001). A one-sample *t*-test also revealed that the RR in treated rats was significantly lower than the theoretical value 0.5 (Fig.3B, dotted line), indicating “no memory” (*P < *0.001). Thus, neurolide-1-treated animals exhibited an improvement in social memory after 2 h compared with untreated animals. The introduction of a new unfamiliar juvenile to the neurolide-1-treated adult animals in the second trial (Fig.3B, white bar) did not lead to a decrease in the RR compared with the vehicle-treated group or with the theoretical value 0.5, indicating that the peptide did not affect social behavior per se, but it had a facilitating effect on memory. Neurolide-1-treated animals did not exhibit an improvement in social memory 24 h after the first trial (Fig.3C).

### Neurolide-1 does not affect spatial learning or long-term memory under normal physiological conditions

We also studied the effect of neurolide-1 on spatial learning and memory in the Morris water maze. We first tested whether the peptide affects memory consolidation. The peptide was injected immediately after the last of the three training sessions in the water maze on days 1 and 2. No effect of neurolide-1 on learning was found on day 2 or 3 or on memory retention in a probe test 24 h after the last training session (Fig. S2). We then changed the administration regimen and injected the peptide 2 h before the training sessions on days 1 and 2. No effect of neurolide-1 on learning, velocity, or memory retention was found in a probe test (Fig. S3). Thus, neurolide-1 affected neither spatial learning nor long-term memory in the Morris water maze.

### Neurolide-1 counteracts MK-801-induced memory deficits in the Morris water maze

We showed that neurolide-1 in neurons induced the phosphorylation of the NMDA receptor NR1 subunit on serine 897 (Fig.1B). We also found that the systemic administration of neurolide-1 counteracted the MK-801-induced inhibition of LTP in acute hippocampal slices (Fig.2). Therefore, we studied whether neurolide-1 attenuates the effect of MK-801 on spatial learning and memory in the Morris water maze. MK-801 (0.1 mg/kg) was previously shown to induce hypermotility (Verma and Moghaddam 1996; Homayoun et al. 2004) and produce a sustained increase in stereotypy that peaked 40 min after injection and returned to baseline 2 h later (Homayoun et al. 2004; Su et al. 2007). In all of our experiments, we used an interval of 2.5–4 h between MK-801 administration and the behavioral tests.

**Figure 4:**
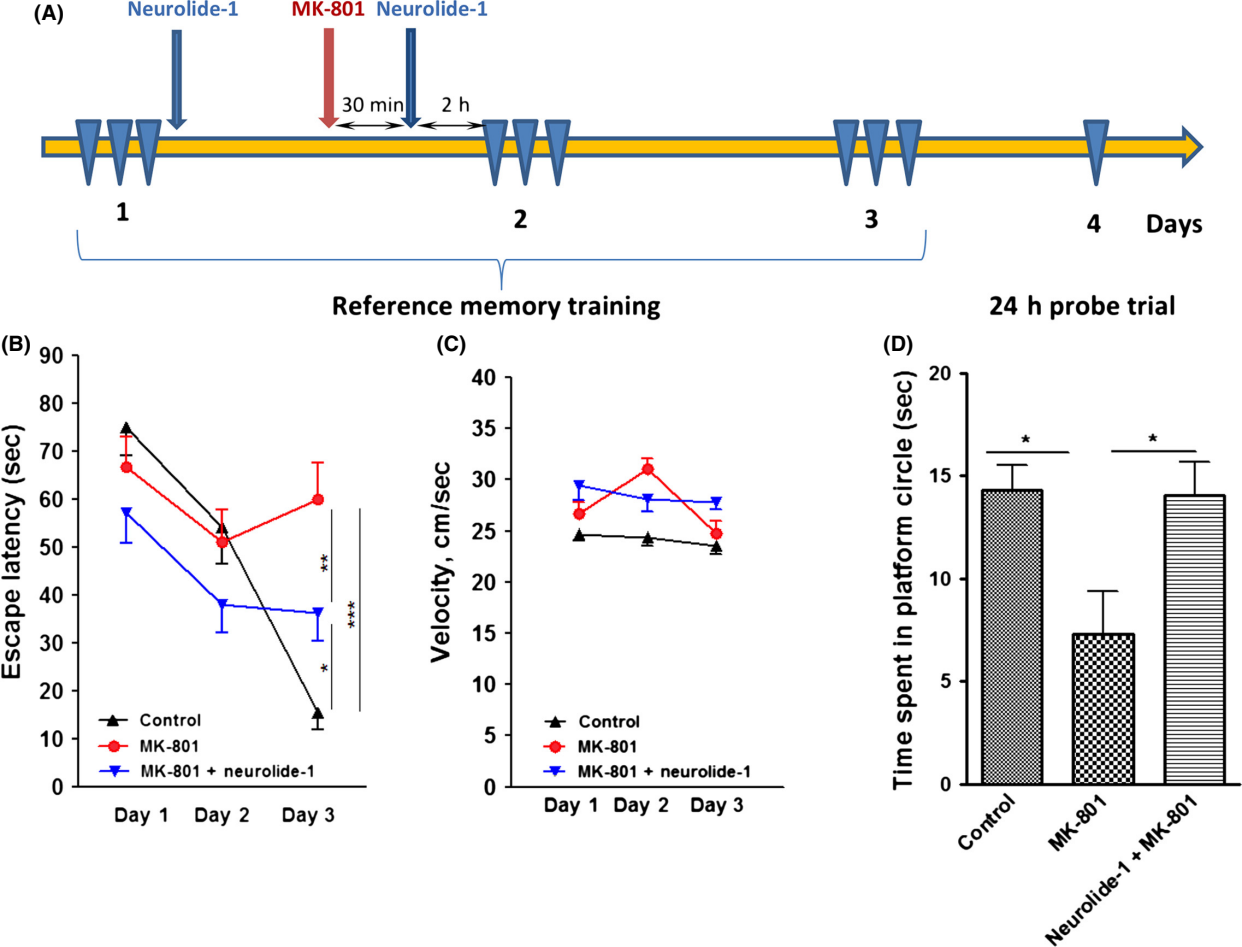
Effect of neurolide-1 on spatial learning and memory in the Morris water maze after treatment with MK-801. (A) Timeline of the experimental paradigm. Reference memory training, escape latency time (B), and velocity (C). Effect on memory retrieval in the probe test (D). The data are expressed as mean ± SEM (*n *=* *12 per group). *P <* *0.05, **P <* *0.01, ***P <* *0.001 (one-way ANOVA followed by Newman–Keuls post hoc test). ANOVA, analysis of variance.

The peptide was injected s.c. on the first day after the training sessions and on the second day, 30 min after MK-801 injection and 2 h before the first training session (Fig.4A). MK-801 strongly inhibited learning. Neurolide-1 attenuated the effect of the NMDA receptor inhibitor on learning (Fig.4B). Neither treatments affected velocity in the water maze (Fig.4C). Neurolide-1 completely abrogated the effect of MK-801 in the probe test 24 h after the last training session (Fig.4D). Thus, neurolide-1 counteracted MK-801-induced learning and memory impairment.

We also studied the effects of MK-801 and neurolide-1 in the Morris water maze when the peptide was injected 30 min after MK-801 and 2 h before the first trial on days 2 and 3 (Fig. S4A). Neurolide-1 did not reduce the effect of MK-801 on learning or memory retention in the probe test (Fig. S4B). Thus, long-term treatment (24 h) with neurolide-1 before MK-801 administration is required to counteract the inhibitory effects of NMDA receptor inhibition on learning and memory.

### Neurolide-1 reduced the effect of MK-801 on working memory in the Y maze

**Figure 5:**
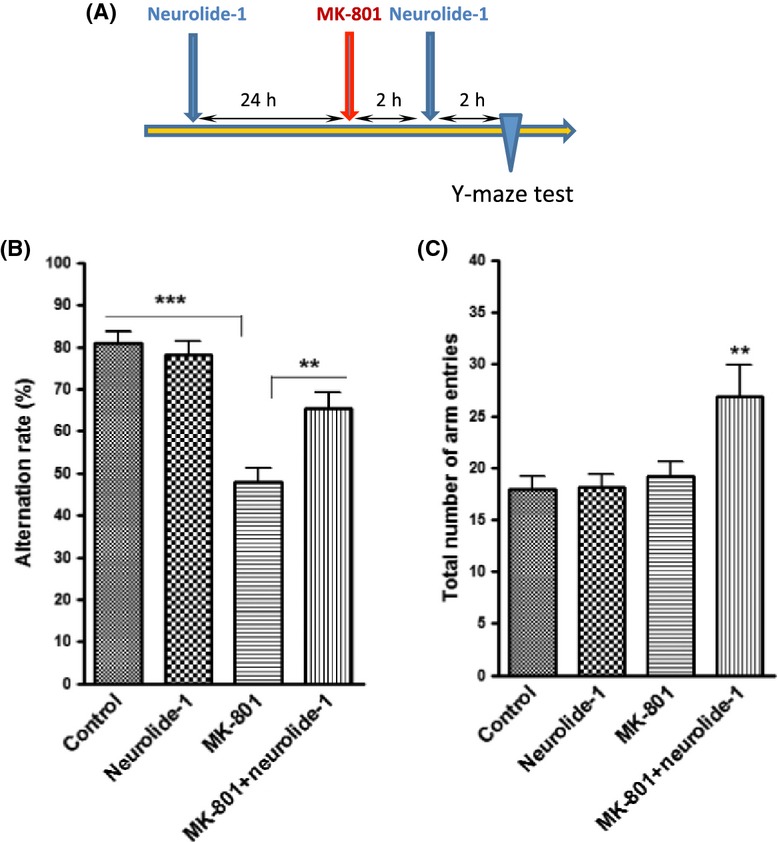
Effect of nerurolide-1 on working memory in the Y maze. (A) Timeline of the experimental paradigm. Alternation rate (B) and number of total arm entries (C). The data are expressed as mean ± SEM (*n *=* *6–12). *P < 0.05, **P < 0.01, ***P < 0.001 (one-way ANOVA followed by Newman–Keuls post hoc test). ANOVA, analysis of variance.

We also investigated whether neurolide-1 counteracts the effect of MK-801 in the Y maze. Adult rats (420–490 g) were treated with the peptide two times, 24 and 2 h before the test. MK-801 was injected 2 h before the second injection of neurolide-1 (Fig.5A). The adult animals all performed well in the Y maze, with an alternation rate between the three arms of ∼80%. Treatment only with neurolide-1 did not affect performance, whereas MK-108 completely impaired working memory, in which the alternation rate was close to 50%, indicating no recollection of visiting the adjacent arms. Neurolide-1 partially restored working memory in MK-801-treated animals (Fig.5B). The total number of arm entries did not change after treatment with the peptide or MK-801 alone, whereas the combined treatment significantly increased the total number of arms visited (Fig.5C).

### Neurolide-1 improved working memory performance in 4-week-old rats in the Y maze

Behavioral abilities, including spontaneous alternation, are well known to not be well-developed in very young rodents (until at least 3 weeks of age), due to the late maturation of anatomical and physiological structures that determine plasticity (Waters et al. 1997). We studied the effect of neurolide-1 on working memory performance in 4-week-old rats (Fig. S5A). At this age, rats practically do not show any preference for entering any arm, with an average alternation rate <60%. A single treatment with neurolide-1 2 h before the test significantly increased the alternation rate to 76% (Fig. S5B). This treatment slightly decreased the total number of arms visited (Fig. S5C).

## Discussion

NL1 is essential for normal excitatory transmission and long-term plasticity. The loss of NL1 expression impairs LTP, reduces synaptic responses upon activation of glutamatergic perforant path granule cell inputs, and reduces the expression of several AMPA and NMDA receptor subunits (Jedlicka et al. 2013). NL1 controls synaptic incorporation and the retention of NMDA receptors through extracellular (*cis*) interactions with the NR1 subunit of the receptor (Budreck et al. 2013). The NL1-binding site for this interaction is unknown. In the present study, we showed that the ectodomain of NL1 that encompasses the unaltered cholinesterase domain and the peptide that corresponds to the NL1 minimal binding site for NX, neurolide-1, both induced phosphorylation of the NMDA receptor NR1 subunit on a serine residue that is known to be involved in receptor trafficking and retention on the surface (Fig.1A and B). This suggests that the neurolide-1 sequence may at least partially represent the NL1-binding site for the NR1 subunit.

The above led us to hypothesize that the previously developed NL1 mimetic peptide, neurolide-1 (Gjorlund et al. 2012), could be a valuable pharmacological tool to facilitate cognitive function, especially cognitive function that is compromised by NMDA receptor inhibition. We found that in vivo administration of the peptide two times, 24 and 1 h before sacrifice, counteracted the MK-801-induced inhibition of LTP in acute hippocampal slices (Fig.2). We began with long-term treatment (24 h) to promote NMDA receptor trafficking before treatment with MK-801, which was also based on our results in which the peptide affected spatial memory (see the Results section).

Although neurolide-1 did not influence spatial learning or memory under normal physiological conditions, it counteracted the effect of the NMDA receptor inhibitor in the Morris water maze in both the learning stage and probe test. Neurolide-1 also partially restored working memory in MK-801-treated animals in the Y maze (Fig.5). Our results clearly indicate that long-term exposure to the peptide (24 h in the present study) is required to observe a neutralizing effect in MK-801-treated animals with regard to both LTP induction and spatial memory. This supports our hypothesis that the mechanism of action of neurolide-1 involves a synaptic increase in NMDA receptors, leading to an increase in the NMDA receptor/inhibitor ratio and restoration of basal synaptic function.

Although neurolide-1 alone had no effect in the Morris water maze, it improved short-term social memory without altering social recognition per se (Fig.3). There is an essential difference between spatial memory observed in rodents in the water maze and social recognition memory. The former is based on visual (spatial) cues, whereas the latter is primarily affected by olfactory and pheromonal cues (Popik et al. 1991). Both types of cognitive behaviors involve functional NMDA receptors. Ionotropic NMDA receptors consist of four subunits, two obligatory NR1 subunits and two NR2A-D or NR3 subunits. In the prefrontal cortex and hippocampus, NR2A- and NR2B-containing receptors are the predominant forms. NR2A- and NR2B-containing receptors have different expression profiles and channel kinetics that likely differentially regulate the behavioral output. Thus, differences in the NR2A:NR2B ratio in various brain structures that are involved in the two types of memories may have determined the observed differences in the effects of the peptide in the water maze and social recognition tests.

Furthermore, the NR2B:NR2A ratio is higher in the juvenile brain after birth when synaptic plasticity is very high compared with adulthood (Jacobs and Tsien 2012). This may partially explain our results from the Y maze test, in which neurolide-1 administration alone did not affect alternation behavior in adult rats but significantly improved working memory in 4-week-old animals (Figs.5 and S5). We suggest that the ability of neurolide-1 to facilitate retention of the NMDA receptor complex is responsible for the observed effect because of the broad window for the regulation of synaptic plasticity in young animals (i.e., high NR2B:NR2A ratio).

The present study identified neurolide-1 as a powerful pharmacological tool to examine the in vivo role of postsynaptic NL1 in cognitive behavior under physiological and pathological conditions. Additionally, our findings emphasize the role of NL1–NMDA receptor interactions in cognition. The effectiveness of neurolide-1 to counteract impairments in cognitive behavior highlights NLs as potential targets for the development of novel drugs that are able to improve cognitive function.

## Abbreviations

aCSF: artificial cerebrospinal fluid
AMPA: *α*-amino-3-hydroxy-5-methyl-4-isoxazolepropionic acid
fEPSP: field excitatory postsynaptic potential
LTP: long-term potentiation
MK-801: (5S,10R)-(+)-5-methyl-10,11-dihydro-5H-dibenzo[a,d]cyclohepten-5,10-imine maleate, Dizocilpine, an uncompetitive antagonist of NMDA receptor
NLs: neuroligins
NMDA: *N*-methyl-d-aspartate
NR1, NR2A, and NR2B: NMDA receptor subunits
NXs: neurexins
PBS: phosphate buffered saline
PKA: protein kinase A
RR: recognition ratios

## Supporting Information

**Data S1.** Supplementary material and methods.

**Figure S1.** Effect of neurolide-1 on LTP in acute hippocampal slices. Neurolide-1 at a concentration of 1 *µ*mol/L was added directly to the running artificial CSF during baseline measurement before the induction of LTP.

**Figure S2.** Effect of neurolide-1 on spatial learning and memory in the Morris water maze. The peptide was injected immediately after the last of the three training sessions on days 1 and 2. Timeline of the experimental paradigm (A). Reference memory training and escape latency (B). Effect on memory retrieval in the probe test (C). The data are expressed as mean ± SEM (*n* = 12 per group).

**Figure S3.** Effect of neurolide-1 on spatial learning and memory in the Morris water maze. The peptide was injected 2 h before the training sessions on days 1 and 2. Timeline of the experimental paradigm (A). Reference memory training, escape latency (B), and velocity (C). Effect on memory retrieval in the probe test (D). The data are expressed as mean ± SEM (*n* = 12 per group).

**Figure S4.** Effect of neurolide-1 on spatial learning and memory in the Morris water maze after treatment with MK-801. Timeline of the experimental paradigm (A). Reference memory training and escape latency (B). Effect on memory retrieval in the probe test (C). The data are expressed as mean ± SEM (*n* = 6–9). ***P *< 0.01, ****P *< 0.001, compared with control (one-way ANOVA followed by Newman–Keuls post hoc test).

**Figure S5.** Effect of neurolide-1 on working memory in 4-week-old rats in the Y maze. Timeline of the experimental paradigm (A). Alternation rate (B) and number of total arm entries (C). The data are expressed as mean ± SEM (*n* = 11–15). **P* < 0.05, ****P* < 0.001.
