# Supplementary figures and images for: A neuroligin-1-derived peptide stimulates phosphorylation of the NMDA receptor NR1 subunit and rescues MK-801-induced decrease in long-term potentiation and memory impairment
Source: Pharmacol Res Perspect. 2015 Mar 13;3(2):e00126. doi: 10.1002/prp2.126 (PMC4448987; doi:10.1002/prp2.126)

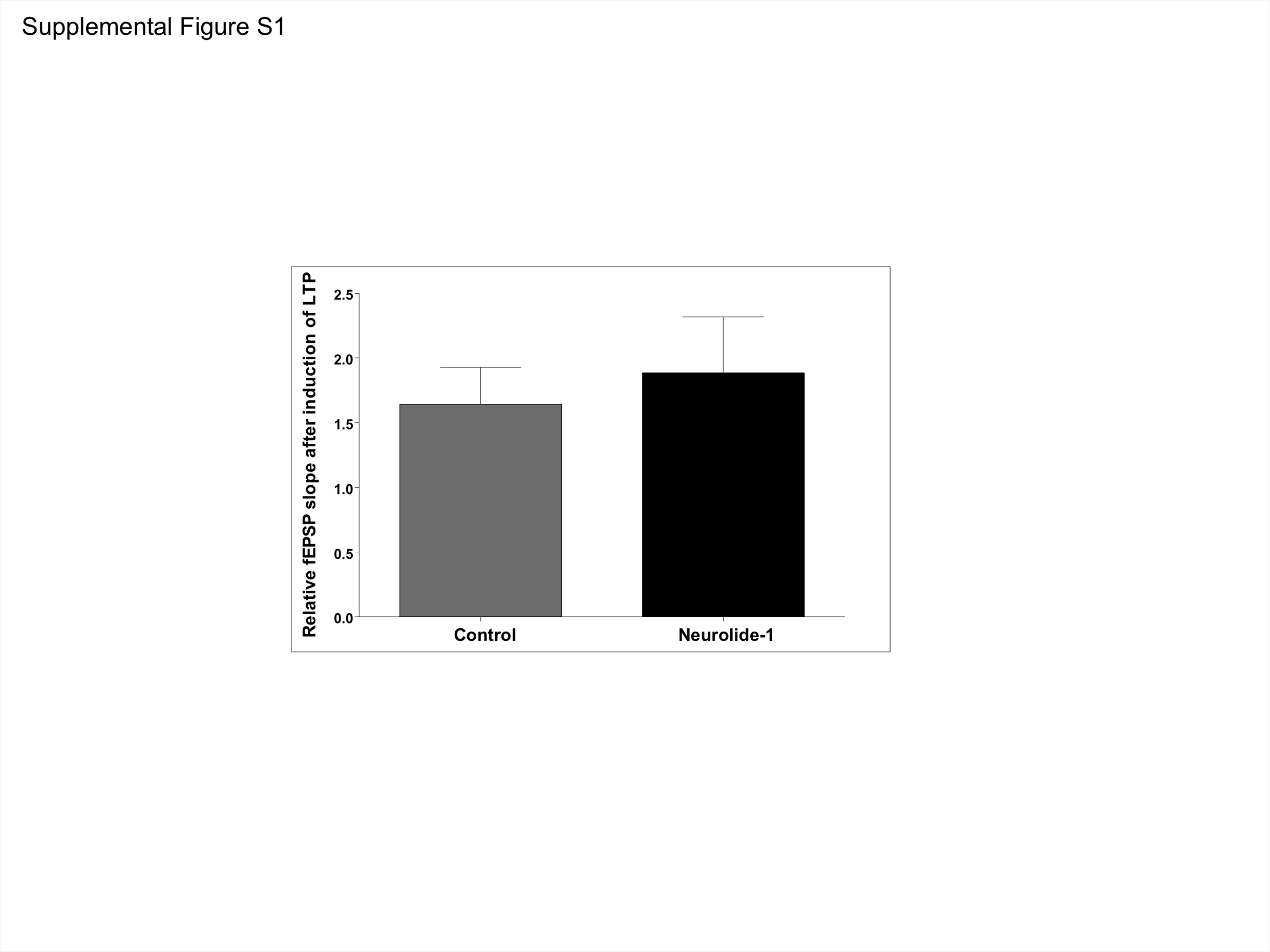

Supplement: Supplementary file 1 — Data S1. Supplementary material and methods. [file prp20003-e00126-sd1.tif]

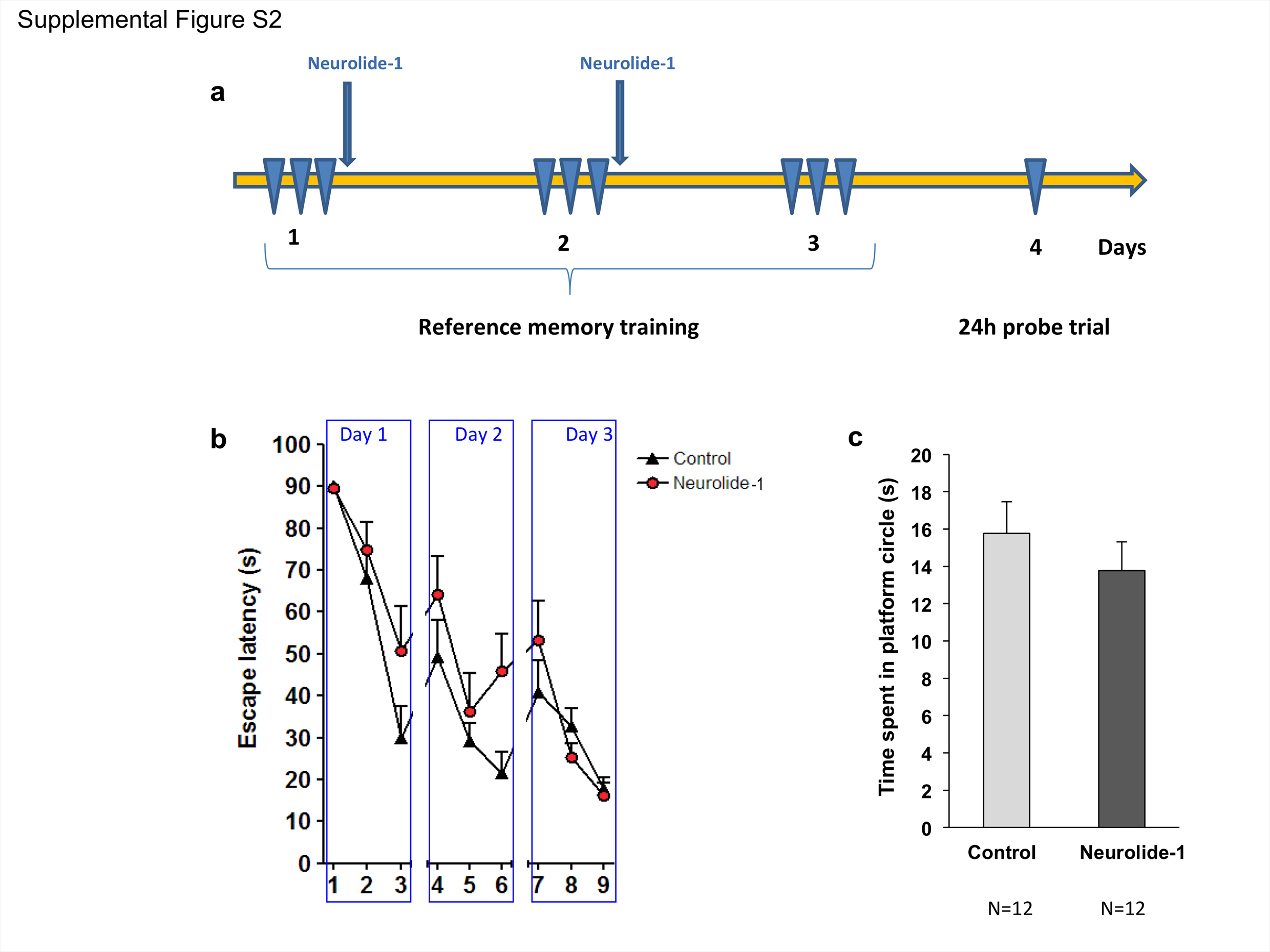

Supplement: Supplementary file 2 — Figure S1. Effect of neurolide-1 on LTP in acute hippocampal slices. Neurolide-1 at a concentration of 1 µmol/L was added directly to the running artificial CSF during baseline measurement before the induction of LTP. [file prp20003-e00126-sd2.tif]

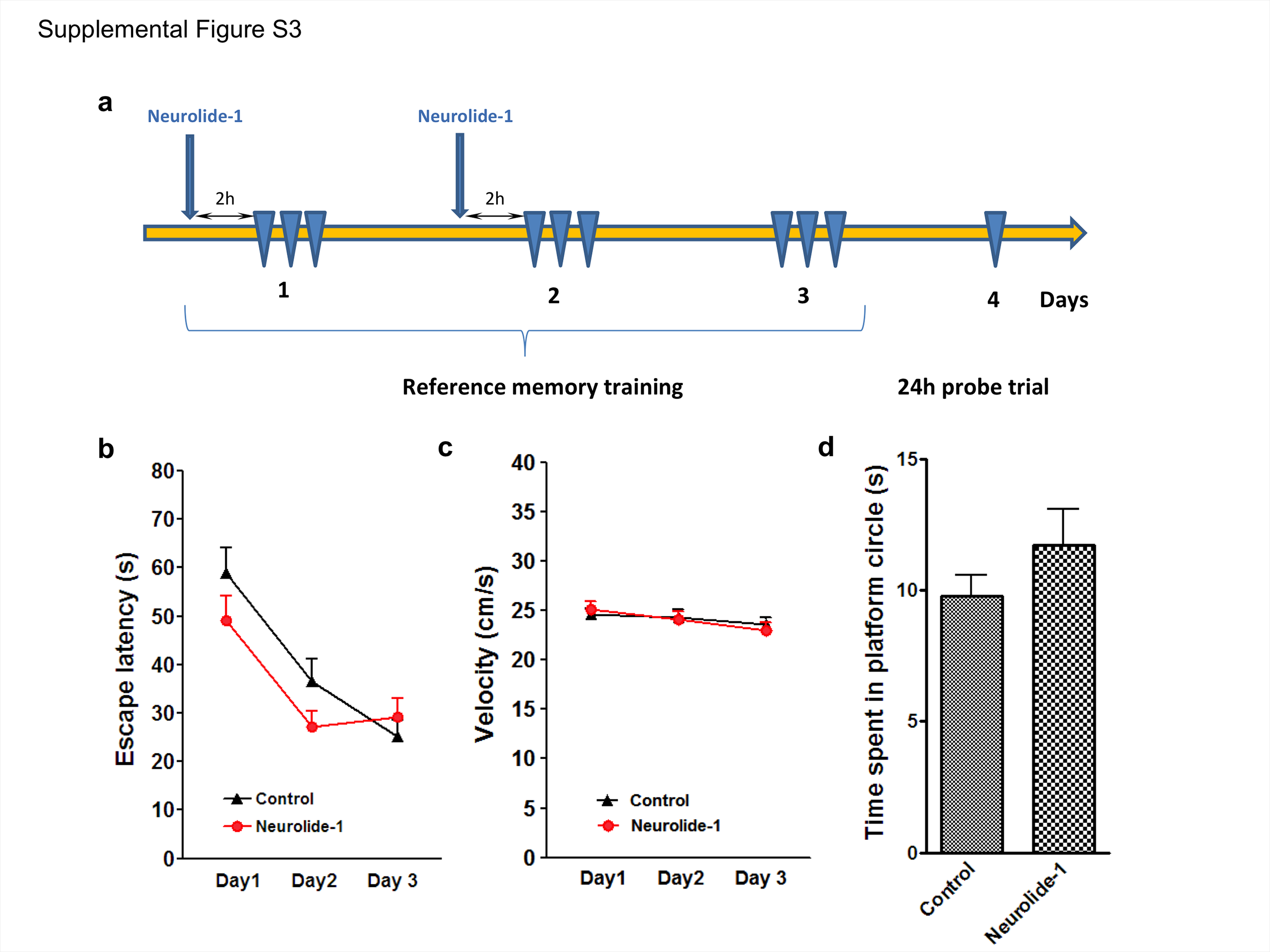

Supplement: Supplementary file 3 — Figure S2. Effect of neurolide-1 on spatial learning and memory in the Morris water maze. The peptide was injected immediately after the last of the three training sessions on days 1 and 2. Timeline of the experimental paradigm (A). Reference memory training and escape latency (B). Effect on memory retrieval in the probe test (C). The data are expressed as mean ± SEM (n = 12 per group). [file prp20003-e00126-sd3.tif]

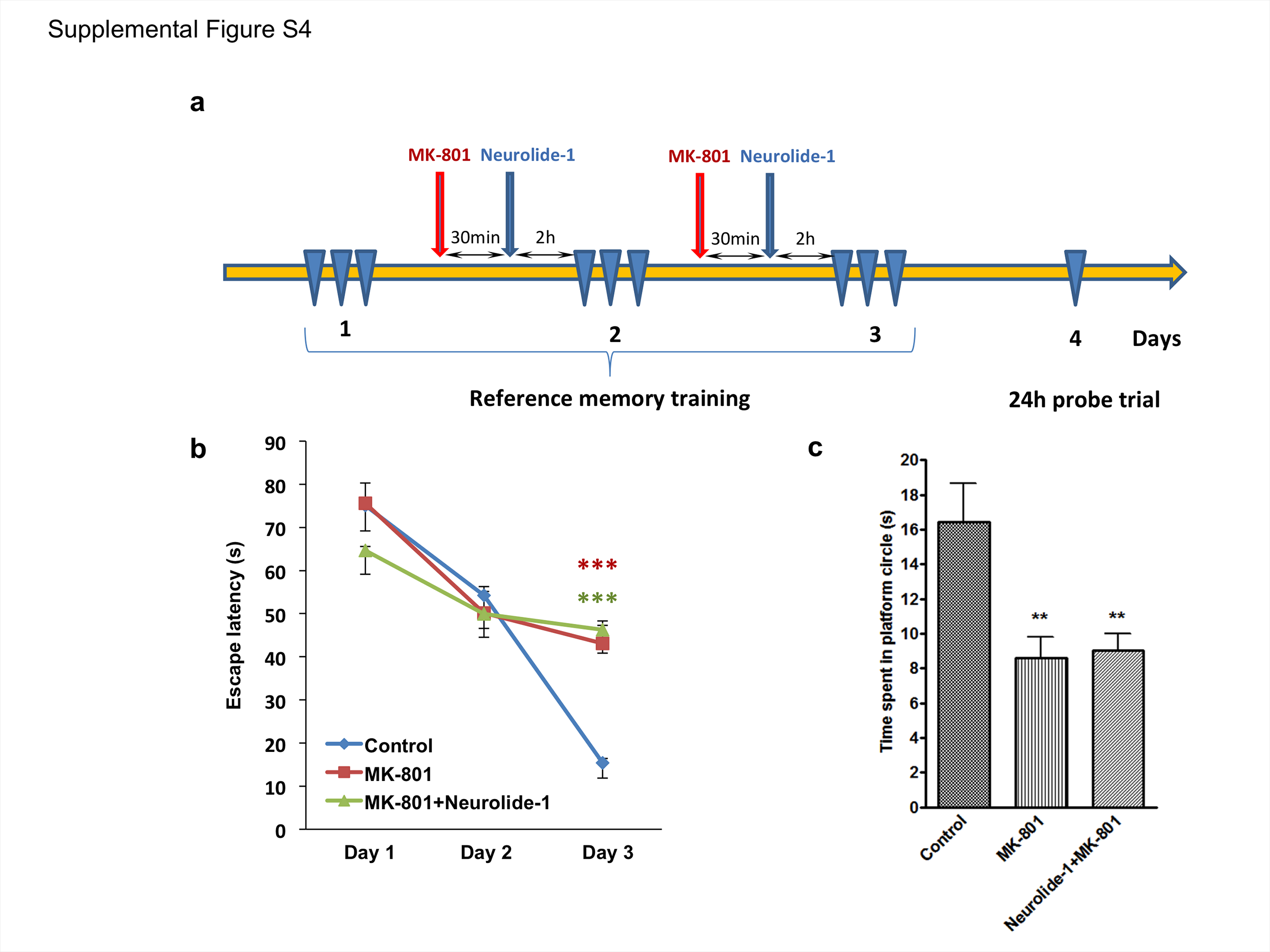

Supplement: Supplementary file 4 — Figure S3. Effect of neurolide-1 on spatial learning and memory in the Morris water maze. The peptide was injected 2 h before the training sessions on days 1 and 2. Timeline of the experimental paradigm (A). Reference memory training, escape latency (B), and velocity (C). Effect on memory retrieval in the probe test (D). The data are expressed as mean ± SEM (n = 12 per group). [file prp20003-e00126-sd4.tif]

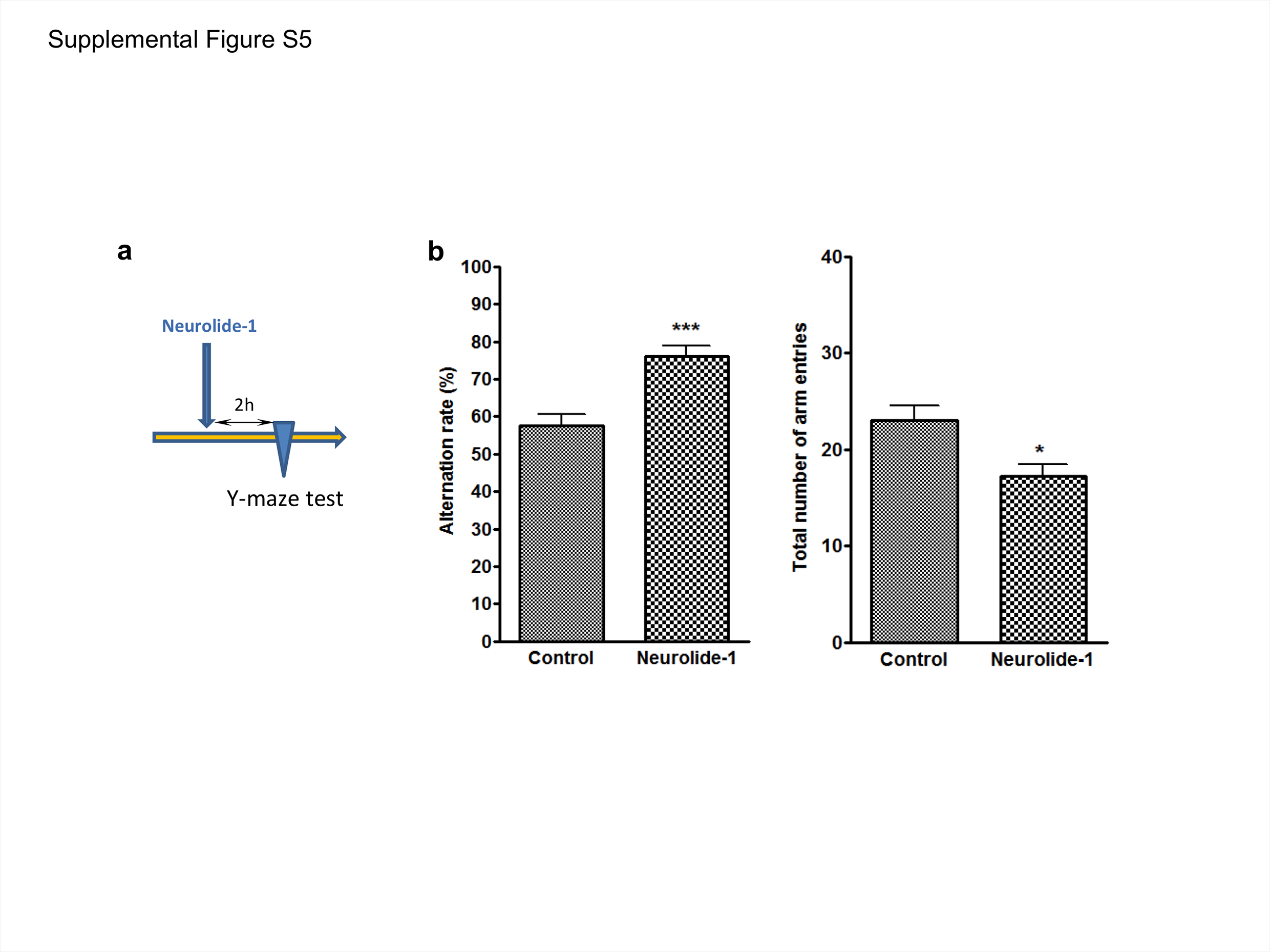

Supplement: Supplementary file 5 — Figure S4. Effect of neurolide-1 on spatial learning and memory in the Morris water maze after treatment with MK-801. Timeline of the experimental paradigm (A). Reference memory training and escape latency (B). Effect on memory retrieval in the probe test (C). The data are expressed as mean ± SEM (n = 6–9). **P < 0.01, ***P < 0.001, compared with control (one-way ANOVA followed by Newman–Keuls post hoc test). [file prp20003-e00126-sd5.tif]
