# Supplementary material for: A neuroligin-1-derived peptide stimulates phosphorylation of the NMDA receptor NR1 subunit and rescues MK-801-induced decrease in long-term potentiation and memory impairment
Source: Pharmacol Res Perspect. 2015 Mar 13;3(2):e00126. doi: 10.1002/prp2.126 (PMC4448987; doi:10.1002/prp2.126)
Supplement: Supplementary file 6 — Figure S5. Effect of neurolide-1 on working memory in 4-week-old rats in the Y maze. Timeline of the experimental paradigm (A). Alternation rate (B) and number of total arm entries (C). The data are expressed as mean ± SEM (n = 11–15). *P < 0.05, ***P < 0.001. [file prp20003-e00126-sd6.docx]

**Supplementary Information**

**SUPPLEMENTARY MATERIAL AND METHODS**

**Peptides**

The neurolide-1 peptide was synthesized using the solid-phase Fmoc protection strategy, and purity was estimated to be ≥ 80% by high-performance liquid chromatography.

**Pharmacokinetics**

The levels of neurolide-1 in plasma and cerebrospinal fluid (CSF) samples were measured by a competitive enzyme-linked immunosorbent assay (ELISA) that was performed on Amino 96-well plates (Nunc, Roskilde, Denmark). The bottom of the wells was coated with biotinylated bovine serum albumin (BSA; Sigma-Aldrich, Brøndby, Denmark) diluted in coating buffer (0.1 M Na-carbonate buffer, pH 9.6) to a concentration of 1 μg/ml, and 100 μl was applied to each well. The plates were incubated overnight at 4°C and then washed three times in washing buffer (phosphate-buffered saline [PBS] with 0.1% v/v Tween-20, pH 7.4). One volume of diluted sample or a standard with a known concentration of the biotin-labeled neurolide-1 was pipetted into the wells of a mixing Protein LoBind Eppendorf Plate (Eppendorf, Hamburg, Germany) and incubated with three volumes of streptavidin-peroxidase (Dako Denmark A/S, Glostrup, Denmark) diluted 1:10,000 in washing buffer. After preincubation for 30 min, 100 μl of the resulting incubation mixture was transferred to the prepared ELISA plate and then incubated for 1 h at 37°C. The plate was then washed three times in washing buffer, and 3,3’,5,5’-tetramethyl-benzidine (TMB) substrate (Kem-En-Tec, Taastrup, Denmark) was added (100 μl/well) and incubated for 5-10 min at room temperature. Color development was terminated by the addition of 2 M H_2_SO_4_ (100 μl/well), and absorbance was read at 450 nm on a Wallac Victor 1420 multilabel counter (PerkinElmer, Hvidovre, Denmark). Peptide concentrations in the samples were determined using a standard curve. Samples obtained from two to six animals were used for each time point and run in duplicate. Two independent determinations were performed for each sample.

**Social recognition test**

Social memory in animals reflects the ability of individuals to recognize conspecifics as familiar or unfamiliar. The animals were handled for 5 days prior to the experiments to minimize stress, habituated to the test cage, and trained to interact with a juvenile rat. Two groups of adult rats were used: a control group and a neurolide-1-treated group. Neurolide-1 (10 mg/kg) or vehicle (saline, 1 ml/kg) was injected subcutaneously (s.c.) two times, 24 and 2 h before the experiment. One hour before the experiment, each animal was housed individually in transparent test cages (25 cm width × 40 cm length × 19 cm height). The test consisted of one trial, a 2 h intertrial interval, and then a test trial. In the first trial, a 3-week-old juvenile male rat was introduced to the experimental adult rat for 4 min, and the time spent investigating the juvenile (e.g., licking, sniffing, chewing, and close following) was scored. Two hours later, a second trial was performed by reintroducing the same juvenile to the test rat to for 4 min, and investigative behavior was recorded. From the two trials, a recognition ratio (RR) was calculated as T2 / (T1 + T2), in which T1 and T2 were the times spent investigating the juvenile during the first and second trials, respectively. The animals were also tested for memory retention 22 h after the second trial (T3). An RR value < 0.5 indicates the retention of social memory. With an intertrial interval longer than 1 h, no or only minimal retention of social memory is expected To verify that any observed effect on social memory was unrelated to non-memory-dependent effects (e.g., locomotion, aggression, and anxiety), a control test was performed under the same experimental conditions, with the exception that an unfamiliar juvenile was introduced in the second trial.

**Spatial memory in the Morris water maze**

The Morris water maze test ([Morris, 1984](#_ENREF_13)) was conducted in a 160 cm wide circular black tank placed in a dimly lit room and filled with 21°C water up to 20 cm from the top. The tank was surrounded by visual orientation marks, and a 10 cm wide escape platform was placed 1.5 cm below the water level for it to be unseen. A video camera was placed above the tank and connected to a computerized tracking system (Ethovision 3.1, Noldus IT, Wageningen, Holland). The tank was divided into four equal-size quadrants that also served as starting positions. The latency to locate the platform, time spent swimming, and swimming speed were scored within the defined zones (i.e., total area, quadrants, and a platform circle that was 60 cm in diameter with the center in the original platform location). Prior to training, the rats were handled 2 min daily for 5 days to minimize stress during the experiments.

Reference memory training consisted of three consecutive trials daily for 3 days. Each trial began with placing the animal in the water facing the wall of the pool. The starting position differed for each trial but was identical for all animals. In each trial, the animal was allowed 90 s to locate the platform. Animals that did not find the platform within 90 s were guided to the platform and given a latency score of 90 s. After each trial, the rats were allowed 20 s of orientation time on the platform and then removed from the pool for 20 s before the next trial was initiated. After the last trial each day, the animals were dried and returned to their home cages.

To test for effects on long-term memory, the animals were tested in a probe trial 24 h after the last reference memory training. In the probe trial, the platform was removed, and the animals began from a position in a quadrant adjacent to the original platform quadrant. The rats were allowed to swim for 60 s, after which the trial was stopped.

**Y maze**

The maze was made of gray polyvinyl chloride, with three identical arms (425 mm length × 225 mm height × 145 mm width) mounted symmetrically to an equilateral triangular center section. Each rat was placed at the end of one arm and allowed to explore the apparatus freely for 8 min. The sequence and number of all arm entries were recorded for each animal throughout the testing period. An alternation was defined as entries into all three arms on consecutive occasions. Therefore, the number of maximal alternation was the total number of arm entries minus 2, and the percentage of alternation was calculated as (actual alternations / maximal alternations) × 100. The total number of arms entered during the sessions was also determined ([Yamaguchi *et al*, 2006](#_ENREF_27)). Data were eliminated in cases in which the number of total arm entries was less than 10 or when the rat escaped from the Y maze. After each trial, the Y maze was cleaned with 1% Virkon S (Pharmaxim, Helsingborg, Sweden).

**Drug treatment**

The neurolide-1 peptide was dissolved in PBS. Neurolide-1 (10 mg/kg) or vehicle was injected subcutaneously (s.c.) in a volume of 1 ml/kg bodyweight. The NMDA receptor antagonist [1]-5-methyl-10,11-dihydro-5H-dibenzo-[a,d]-cyclohepten-5,10-imine hydrogen maleate (MK-801; Sigma-Aldrich, Brøndby, Denmark) was dissolved in PBS. MK-801 (0.1 mg/kg) or vehicle was injected s.c. in a volume of 1 ml/kg body weight. This dose has been previously reported to impair cognition in rats without causing significant sensory, locomotor, or toxicological side effects and reaches a sufficient level of extracellular fluid concentrations in the brain to disrupt NMDA receptor function.

**SUPPLEMENTARY FIGURE LEGENDS**

**Supplementary Figure S1.** Effect of neurolide-1 on LTP in acute hippocampal slices. Neurolide-1 at a concentration of 1 µM was added directly to the running artificial CSF during baseline measurement before the induction of LTP.

**Supplementary Figure S2.** Effect of neurolide-1 on spatial learning and memory in the Morris water maze. The peptide was injected immediately after the last of the three training sessions on days 1 and 2. Timeline of the experimental paradigm **(a)**. Reference memory training and escape latency **(b)**. Effect on memory retrieval in the probe test **(c)**. The data are expressed as mean ± SEM (*n* = 12 per group).

**Supplementary Figure S3.** Effect of neurolide-1 on spatial learning and memory in the Morris water maze. The peptide was injected 2 h before the training sessions on days 1 and 2. Timeline of the experimental paradigm **(a)**. Reference memory training, escape latency **(b)**, and velocity **(c)**. Effect on memory retrieval in the probe test **(d)**. The data are expressed as mean ± SEM (*n* = 12 per group).

**Supplementary Figure S4.** Effect of neurolide-1 on spatial learning and memory in the Morris water maze after treatment with MK-801. Timeline of the experimental paradigm **(a)**. Reference memory training and escape latency **(b)**. Effect on memory retrieval in the probe test **(c)**. The data are expressed as mean ± SEM (*n* = 6-9). ***p*< 0.01, ****p*< 0.001, compared with control (one-way analysis of variance followed by Newman-Keuls *post hoc*test).

**Supplementary Figure S5.** Effect of neurolide-1 on working memory in 4-week-old rats in the Y maze. Timeline of the experimental paradigm **(a)**. Alternation rate **(b)** and number of total arm entries (c). The data are expressed as mean ± SEM (*n* = 11-15). **p* < 0.05, ****p* < 0.001.
